# Production and characterization of nondairy gluten‐free fermented beverage based on buckwheat and lentil

**DOI:** 10.1002/fsn3.3095

**Published:** 2022-10-20

**Authors:** Mir‐Hossein Mousavi, Mehdi Gharekhani, Kazem Alirezalu, Leila Roufegarinejad, Sodeif Azadmard‐Damirchi

**Affiliations:** ^1^ Department of Food Science and Technology Tabriz Branch, Islamic Azad University Tabriz Iran; ^2^ Department of Food Science and Technology, Ahar Faculty of Agriculture and Natural Recourses University of Tabriz Tabriz Iran; ^3^ Department of Food Science and Technology, Faculty of Agriculture University of Tabriz Tabriz Iran

**Keywords:** buckwheat, fermented beverage, gluten free, lentil, probiotic

## Abstract

The present study aimed to optimize the formulation of buckwheat/lentil gluten‐free beverages fermented with *Lactobacillus plantarum* and *Bifidobacterium bifidum*. Physicochemical parameters of 14 different beverages, such as pH, acidity, total solids, ash, total phenol content, antioxidant activity, and sensory test, were assessed after 24 h of fermentation. The results showed that the numbers of viable cells of lactobacilli and bifidobacteria on the first day of the experiment were 9.9 and 9.6 log (CFU ml^−1^), respectively, which were over 9 log (CFU ml^−1^). During 24 h from the fermentation, the number of viable cells for all beverages decreased, which reached an average probiotic count of 8.81 log (CFU ml^−1^) that was statistically significantly different from the probiotic count before fermentation (*p* < .05). Cell viability was evaluated and shelf life was estimated during 15‐day refrigerated storage. At the end of the storage (15th day), the beverages contained an average of 8.4 log (CFU ml^−1^) of live lactobacilli cells and 7.8 log (CFU ml^−1^) of viable bifidobacterial cells. The optimized levels of independent factors for sprouted buckwheat and lentil flours were 51.96% and 48.04%, respectively. The optimized probiotic beverage was contained 0.25 (% lactic acid) acidity, 5.7 pH, 7.9% total solids, 0.4% ash, 41.02% DPPH, 26.96 (mg GAE/ml) phenol compounds, and 8.65 log (CFU ml^−1^) probiotic count. The optimized beverage had distinct organoleptic properties on day 15 of refrigerated storage. This study showed that *Bifidobacterium bifidum* can be used for the development of potentially probiotic beverage with sprouted buckwheat and lentil.

## Graphical abstract

Physicochemical parameters of 14 different beverages were assessed by passing 24 hours from the fermentation. This study showed Bifidobacterium bifidum can be used for the development of potentially probiotic beverage with sprouted buckwheat and lentil.

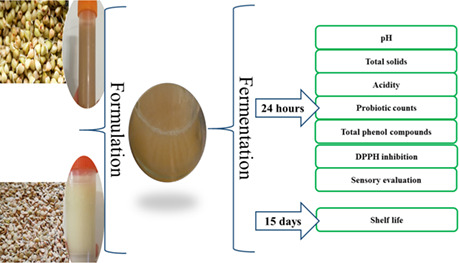

## 1 INTRODUCTION

Fermented beverages have progressed from traditional natural fermented products to those beverages formulated with functional ingredients, which are used to stimulate cardiovascular benefits, and then to ferment drinks that improve gastrointestinal health and can further evolve to fermented foods designed with specific bioactive nanoparticles (Salmerón, 2017). Currently, the development of novel functional beverages is a growing sector of food industry due to the increased demand for foods that donate health benefits and consequently reduce the risk of the development of human diseases (Yépez et al. 2019). The use of nondairy probiotic beverages is attractive due to avoiding dairy allergens and intolerances, reducing cholesterol and saturated fatty acids intake, and supporting vegetarians' lifestyles (Salmerón 2017). These serve as a cost‐effective alternative to the impoverished sector of developing countries and in areas with limited milk supplies (Chaturvedi and Chakraborty 2021). In patients with celiac disease, immune responses to gliadin fractions promote an inflammatory reaction, primarily in the upper small intestine, characterized by the infiltration of the lamina propria and the epithelium with chronic inflammatory cells. The absence of gliadin and glutenin complex (gluten) in buckwheat (*Fagopyrum esculentum*, L.) is another reason of the noticeable rise in the use of this grain for functional beverages (Gimenez‐ Bastida et al. 2015b). Buckwheat is a rich source of starch; gluten‐free protein; vitamins such as thiamine, riboflavin, niacin, pantothenic acid, and pyridoxine; and minerals in higher amounts than in conventional cereals and antioxidants such as rutin, orientin, vitexin, quercetin, isovitexin, and isoorientin. Due to its potential health benefits, buckwheat and buckwheat‐based products like pseudocereal probiotic beverages have been receiving increasing attention as a functional food (Ugural and Akyol 2020). Legumes are known as an excellent source of protein, complex carbohydrates, dietary fiber, essential vitamins and minerals (Mamilla and Mishra 2017), and natural antioxidants (Dhull et al. 2020). However, the presence of antinutritional factors, such as tannins, phytates, trypsin inhibitor, and hemagglutinin, limits the use of legumes as a basic source of protein (Semba et al. 2021). Germination is known as one of the most commonly used approaches to reduce most of the antinutritional factors of legumes (Mamilla and Mishra 2017). According to the available literature, during germination of legumes, water absorption capacity, protein solubility index, emulsification capacity, and protein and carbohydrate digestibility (Mamilla and Mishra 2017) improve, and vitamin contents and their bioavailability increase as well (El‐Adawy et al. 2003).

Lentils (Lens culinaris L.) are the most available and consumed type of legumes worldwide. Lentil is rich in fibers; resistant starch; oligosaccharides; proteins; vitamins (Mamilla and Mishra 2017); and minerals such as magnesium, phosphorus, calcium, sulfur (Paucean et al. 2018), Iron, zinc, copper, calcium, and magnesium (Ramírez‐Ojeda et al. 2018).

Lentils have a good amount of protein, sugars, and dietary fiber and majorly help to fight against heart disease and cancer (Chaturvedi and Chakraborty 2021).

Several studies have previously reported that the presence of phytochemicals such as polyphenols, phytosterols, and bioactive peptides, as well as other related phenolic compounds, make lentils a suitable functional food with several antioxidant and antidiabetic properties (Dhull et al. 2020). The commercial nondairy beverages widely produced are originated from a variety of plant‐derived ingredients such as cereals, millets, legumes, pseudocereals, nuts, fruits, and vegetables.

The potential of lentil to be employed as nondairy milk was also explored by Zare et al (2011, 2013).

There are several works on formulating beverages from legumes and cereals, the effect of probiotic on the beverage, its viability, shelf life of the product, etc. However, such beverages can also be developed using a combination of legumes and pseudocereals like buckwheat. Therefore, lentils and buckwheat could be known as a suitable matrix for probiotics. They can stimulate the growth of single‐ and mixed‐culture fermentations of probiotic microorganisms. Fermentation in plant‐based milk substitutes allows better sensory and nutritional properties together with enhanced shelf life (Chaturvedi and Chakraborty 2021).

This study aimed to formulate a novel fermented buckwheat/lentil‐based beverage and to optimize the formulation of beverages. Additionally, buckwheat/lentil‐based fermented beverages were studied for their biochemical and nutritional compositions and viable cell count during 15 days of refrigerated storage.

## 2 MATERIAL AND METHODS

### 2.1 Raw materials/reagents

LABs used in the present study were one Lactobacillus Plantarum strain (ATCC 14917), and one Bifidobacterium bifidum (ATCC 29521) purchased from the Iran Industrial Microorganism Collection (Biotechnology Research Institute of the Iranian Scientific and Industrial Research Organization (IROST)). Moreover, Folin–Ciocalteu's phenol reagent and DPPH (2,2‐diphenyl‐1‐picrylhydrazyl) were purchased from Sigma Aldrich. High fructose corn syrup (HFCS) (SKU: HFCS42‐P) was supplied by a Starch & Sweetener producer (FFNAB company, Tehran, IRAN). All other reagents used in the experiments were of analytical grade.

### 2.2 Preparation of flours

Fresh lentil and buckwheat were purchased from a local market (Refah, Tabriz, IRAN). Firstly, Lentil and buckwheat grains were separately cleaned. Thereafter, 500 grams of each grain was soaked in water for 20 h. In order to disinfect and accelerate the germination process, grains were placed in sodium hypochlorite (2%) solution for 10 min, washed with distilled water, and placed in a damp cloth for 72–120 h until sprouting the grains. Finally, the sprouted grains were dried at room temperature and ground using a laboratory mill. Subsequently, the sprouted lentil flour and sprouted buckwheat flour were used as substrates for submerged fermentation (Sharma et al. 2014).

### 2.3 Probiotic cultures

The probiotic strains, namely, *L. plantarum* (ATCC 14917) and *B.bifidum* (ATCC 29521), were maintained at 4°C and then subcultured monthly on slants prepared from MRS (de Man, Rogosa, and Sharpe) agar. The activation of microbial culture and extraction of the pellet were done according to Sharma et al. (2014). Culture was activated in MRS broth by transferring 0.1 mg of freeze‐dried culture in 10 ml of MRS broth and the tube was incubated at 37°C for 24–48 h. From this 10 ml, 1 ml was taken in 100‐ml MRS broth and this culture was reactivated at 37°C for 24–48 h with several transfers of the culture. Finally, 1 ml from the last 10 ml was taken in 100‐ml MRS broth and incubated at 37°C for 24–48 h. A quantity of 1 ml of the activated culture was placed on MRS agar at 37°C for 48 h. Then, colonies were chosen and gram staining was done. Rod‐shaped pink‐colored colonies were observed under microscope and these were picked and their growth was observed in MRS broth at 37°C for 24–48 h.

### 2.4 Selection of starters

MRS (de Man, Rogosa, and Sharpe, which is a selective medium for the isolation of lactic acid bacteria) agar (Merck, Darmstadt, Germany) was used to determine the number of lactobacilli cells and BSM agar (*Bifidus* Selective Medium Agar, Sigma‐Aldrich, St. Louis, MO, USA) was applied to determine the number of *bifidobacteria* cells. Afterward, microbiological analysis was performed using the plate count method. The media were incubated for 72 h at 37°C to produce visible well‐separated colonies. Hence, each strain was separately inoculated (in duplicate) into the formulated fermentation substrates prepared with lentil and buckwheat (according to Table 1). For each strain, an aliquot of the bacterial biomass was inoculated with a sterile disposable loop (1 μl) under the sterilized conditions to an initial cell density of ~8 log colony‐forming units (CFU) ml^−1^. Cell viability and load were checked immediately after the inoculation through plate count method on MRS agar (VWR) (Cardinali et al. 2021).

**TABLE 1:** Experimental design of the levels, variables of D‐optimal, and beverage properties

Run	Independent variable			Responses							
	X1	X2	X3								
	Buckwheat (%)	Lentil (%)	LAB	pH	Acidity (% lactic acid)	Total solids (%)	Ash (%)	Acceptance score	DPPH (%)	Phenol Compounds (mgGAE/ml)	Probiotic Counts (log_10_ CFU ml^−1^)
1	67.66	33.33	*L.plantarum*{−1}	5.20	0.16	7.60	0.36	3.30	42.63	28.23	8.94
2	100	0	*L.plantarum*{−1}	5.20	0.18	8.00	0.41	2.90	40.23	26.36	8.79
3	100	0	*B. bifidum* {1}	5.70	0.25	7.90	0.4	3.10	41.02	26.96	8.65
4	50	50	*B. bifidum* {1}	5.30	0.32	7.50	0.32	4.20	44.12	31.3	8.93
5	100	0	*B.bifidum* {1}	5.20	0.18	7.90	0.39	3.00	38.19	24.36	8.66
6	75	25	*B. bifidum* {1}	5.00	0.35	8.10	0.39	3.10	39.63	25.36	8.88
7	0	100	*B. bifidum* {1}	5.90	0.11	7.10	0.32	3.80	45.33	32.36	8.35
8	0	100	*L.plantarum*{−1}	5.10	0.19	7.00	0.31	3.60	46.32	34.23	8.92
9	75	25	*L.plantarum*{−1}	5.25	0.18	7.70	0.38	3.10	38.21	28.36	8.93
10	25	75	*B. bifidum* {1}	5.10	0.26	7.10	0.35	4.30	44.23	31.02	8.86
11	0	100	*L.plantarum*{−1}	5.15	0.19	7.00	0.32	3.30	45.32	33.12	8.81
12	25	75	*L.plantarum*{−1}	5.25	0.17	7.20	0.33	3.90	46.52	33.02	8.96
13	50	50	*L.plantarum* {−1}	5.20	0.21	7.50	0.34	4.10	40.12	30.23	8.92
14	0	100	*B. bifidum* {1}	5.70	0.24	7.00	0.33	4.00	44.23	34.25	8.74

### 2.5 Experimental design

Experimental D‐optimal design of the two mixture's components (Buckwheat and lentil), one categorical factor with two levels (as *L. plantarum* and *B. bifidum*) (Table 2) and beverage properties with point exchange mode consisting of eight points, two additional center points, and five replications, were used in this study. The combined study was applied to investigate the effect of lentil and buckwheat concentrations and type of LAB on the chemical properties and the survival of LAB of the fermented beverages (Table 2) using design expert (Version 5.0.9) software (Stat‐Ease Corporation, USA). Analysis of variance was then used for the evaluation of the combined (Mean × Main) effects. The model and final equations were applied in terms of L_Pseudo Coding and coded for categorical factors.

**TABLE 2:** Levels of mixture factors, categoric factors, and the constraint

		Low level	High level
Mixture factors	X1 = Buckwheat (%)	0	100
	X2 = Lentil (%)	0	100
Constraint	A + B = 100		
Categoric factor	X3 = LAB		

### 2.6 Production of prototypes of the fermented beverages

For preparing the fermented beverage samples, the obtained flour of each grain was mixed with water‐to‐flour ratio of 5:1 (w/w) and then maintained for 24 h at room temperature. The samples were centrifuged for 20 min at 400 rpm and the supernatant was used to prepare the prototypes of fermented beverages according to Table 1 and then mixed with 5% HFCS and pasteurized for 5 min at 90°C (Sharma et al. 2014). The obtained samples were cooled to 37°C, and microbial cell pellets were added to them at an initial concentration of 1% (v/v) according to Table 1. Next, the samples were incubated for 24 h at 37°C and then stored at the refrigerator (4°C) for further experiments. The flour of lentil and buckwheat was used as control sample.

### 2.7 Characterization of beverage

Aliquots of the prototype fermented beverages were sampled under sterile conditions and analyzed for pH and total titratable acidity (TTA), total phenol content, antioxidant activity (DPPH%), total solids, and ash. After 24 h of fermentation, three independent measurements were performed for each sample and the results were expressed as mean value ± standard deviation. For determining the number of each type of bacteria, three repetitions were made and the microbiological counting was performed after 24 h of fermentation until the 15th day of fermentation in 3‐day intervals of refrigerator storage. For evaluating the sensory properties, special forms were filled by 20 panelists during 15 days with a 3‐day interval.

### 2.8 Determination of pH and total titratable acidity (TTA)

The pH value was determined using a pH meter (Model 300, Hanna Instruments, Padova, Italy) equipped with a solid electrode (Model HI2031, Hanna Instruments). Total titratable acidity (TTA) was determined as described in the study by Cardinali et al. (2021). For measuring TTA, 10 grams of each sample was homogenized with 90 ml of distilled water, and TTA was determined as the volume of 0.1 M NaOH, used to obtain a pH value of 8.3 (Cardinali et al. 2021).

### 2.9 Measurement of total solids

The total solid value of samples was calculated by refractive index using ABBE precision refractometer (Atago, 3 T Model, Honshu, Japan) (Kaashyap, et al. 2021).

### 2.10 Measurement of ash value

Ash content of the fermented beverages was determined using the analytical references, previously reported in the study by Wang et al. (2018).

### 2.11 Antioxidant activity

The DPPH‐scavenging activity of sample was analyzed by adapting the method described by Dhull et al. (2020). For this purpose, 100 μl of sample was taken in spectrophotometric cell and then 3 ml of 100‐μM DPPH was added. The changes in absorbance at 517 nm were recorded after 30 min. Percent (%) DPPH‐scavenging activity was calculated using the following

formula: DPPH‐Scavenging Activity % = (A_c_ − A_E_/A_c_) × 100, where A_C_ and A_E_ are the absorbance of control and extracts, respectively.

### 2.12 Total phenol content

The total phenol content of extracts was measured using Folin–Ciocalteu's colorimetric method. Accordingly, 0.5 ml of Folin–Ciocalteu reagent (0.2 N) was added to 0.5 ml of the methanol extract (prepared by dissolving 10 mg of sample in 30 ml of methanol) and incubated for 3 min at room temperature. After the addition of 10 ml of sodium carbonate solution (75 g/L) and 5‐ml distilled water and mixing them together, the samples were incubated for 1 h at room temperature in a dark place. The absorbance rate was measured at 750 nm and TPC was expressed as gallic acid equivalents. As well, gallic acid was used in the range of 0–100 mg to produce a standard calibration curve (Mamilla and Mishra 2017).

### 2.13 Microbiological analyses

In regard to the viable counting of lactic acid bacteria, an aliquot of each analyzed sample (1 or 10 g) was accurately homogenized in sterile peptone water (bacteriological peptone,0.1% w/w) at a 1:9 (w/ v) ratio using a stomacher apparatus for 1 min at 260 RPM. Subsequently, serial decimal dilutions were prepared with the same diluent and 100 μl of each dilution was inoculated in duplicate using streak‐plate technique on MRS agar and BSM agar and then incubated for 48–72 h at 30°C in anaerobic jars using AnaeroGen 2.5 L Atmosphere Generation Systems (Thermo Scientific, Massachusetts, USA). Plates showing 30–300 colonies were used for the enumeration. The obtained results were expressed as the mean log (CFU ml^−1^) of three replicates ± standard deviation (Cardinali et al. 2021).

### 2.14 Evaluation of media selectivity

Twenty colonies per each medium were selected for further confirmatory tests. MRS medium with vancomycin (pH 5.6) and cultivation under aerobic conditions at 30°C was used for selective enumeration of *L. plantarum* after cultivation in broth, then the purity of *L.plantarum* cultures on this medium was monitored by colony morphology, Gram staining, and microscopy (Olympus CX23; Olympus, Tokyo, Japan) (Veselá et al., 2019).

Pure isolates of *B.bifidum* were cultivated in Wilkins–Chalgren broth supplemented with soya peptone (5 g/L, Oxoid). Tests were conducted for morphology, Gram staining, and fructose‐6‐phosphate phosphoketolase (specific enzyme for Bifidobacteriaceae family) activity (F6PPK test) in order to confirm the selectivity of the BSM agar for bifidobacteria (Bunesova et al., 2015).

### 2.15 Shelf life evaluation

The shelf life of the fermented lentil and buckwheat beverages was defined as the period of cold storage (4°C), during which pH value remained above 4.0 and the number of viable cell counts was above 10^6^ CFU ml^−1^. Cold storage was performed for 15 days with the periodical observation of pH and viability of starter culture, which was performed for the cold storage (4°C) every 3 days for a period of 15 days (Verni et al. 2020).

### 2.16 Sensory properties

The sensory properties of beverages, such as taste, smell, color, mouthfeel, and general acceptability, were evaluated by 20 panelists. Scores were given based on a hedonic scale of 1 to 9 as follows: 1 = dislike very much (very bad) and 9 = like very much (excellent) (Hassan et al. 2012).

### 2.17 Statistical analysis

All these experiments were done in triplicates. The optimization was conducted using Design‐Expert software, version 10 (Stat‐Ease, Inc, Minneapolis, US). Normal distribution and homogeneity of variance were previously examined (Shapiro–Wilk). Significantly different data were indicated with a different superscript letters.

## 3 RESULTS AND DISCUSSION

### 3.1 Chemical composition of buckwheat and lentil flours

The results of the composition analysis of grain flour showed the total solid value of buckwheat and lentil flours as 96.8% and 97.2%, respectively. The dietary fiber content of buckwheat flour was 7% dry matter (dm). This result is in agreement with the value of 7% obtained for the dietary fiber content of buckwheat (Gimenez‐Bastida and Zielinski 2015).

The dietary fiber content of lentil flour was 13.43% (dm), which is close to the value of 14% previously reported for the dietary fiber content of lentils (Araya et al. 1990). This result suggests that these grains could be recognized as a good source of nutritionally valuable dietary fiber (Gimenez‐Bastida and Zielinski 2015).

The pH and acidity values of buckwheat flour were calculated as 6.25 (% lactic acid) and 0.08, respectively. The pH value of lentil flour was 6.51, which is close to the value (6) determined for pH in the nonfermented lentil flour by Oliveira and Castro (2020). In addition, the acidity value of lentil flour was 0.06 (% lactic acid), which differs from the value of 1.14% (% lactic acid) for the acidity value of raw lentil reported in the study by Frias et al. in 1996.

### 3.2 Optimization and fitting the model

At this stage, both modeling and optimization of the properties of the fermented beverages were dependent on buckwheat and lentil concentrations and LAB activity. RSM was used for beverage properties' modeling and the optimal conditions' determination for beverage formulation. Next, the optimization of the formulation variables was done using a combined D‐optimal design. Numerical optimization technique of Design Expert was used for simultaneous optimization of the multiple responses. The desired goals for each factor and response were chosen. The possible goals were maximize, minimize, target, within range, equal to (for factors only). All the independents factors were kept within the experimental range while the responses were either maximized or minimized. In order to search a solution for multiple responses, the goals were combined into an overall composite function which is called the desirability function. Desirability is an objective function that ranges from zero (least desirable) outside the limits to one (most desirable) at the goal. The numerical optimization finds a point that maximizes the desirability function. The goal seeking begins at a random starting point and proceeds up the steepest slope to a maximum. Numerical optimization technique of Design Expert was used for simultaneous optimization of the multiple responses. Accordingly, for all factors including buckwheat, lentil, and LAB we chose the goal of in range while for the responses including acidity, total solids, ash, probiotic counts, total phenol, and acceptance score, we chose to maximize goal and for the pH response the goal was chosen to be minimized.

The properties of the selected model for beverage attributes are presented in Table 3. The result of analysis of variance of the response surface linear mixture model for pH is given in Table 3. The F value of 4.14 and *p*‐value smaller than .03 indicate that the model for pH was significant. It is evident from the results that a significant effect of buckwheat and lentil concentrations exists on the pH values of the probiotic beverage. The first‐order linear mixture model was obtained by model fitting for pH as the function of sprouted buckwheat, sprouted lentil, and LAB as: pH = 0.32X_2_X_3_–1.20 X_1_X_2_X_3_. The results of the analysis of variance for the response surface linear model for acidity are given in Table 3. The R^2^, adj R^2^, and CV values were obtained as 0.72, 0.55, and 20.11, respectively, indicating that the model can be used to navigate the design space. The first‐order linear model was obtained using the model fitting for acidity as the function of sprouted buckwheat, sprouted lentil, and LAB as: Acidity = 0.27X_1_X_2_ + 0.27X_1_X_2_X3. The results of the analysis of variance for the response surface linear model for total solids are given in Table 3. The R^2^, adj R^2^, and CV values were obtained as 0.99, 0.98, and 0.74, respectively. The value of R^2^ implied that this model could explain a high percentage of the variation in the observed data. The first‐order linear model was obtained using the model fitting for total solids as the function of sprouted buckwheat, sprouted lentil, and LAB as: Total solids = 1.49 X_1_X_2_(X_1_‐X_2_) + 1.58 X_1_X_2_X_3_(X_1_‐X_2_). The linear mixture model for the ash content, DPPH, and total phenol was significant (Table 3) although analysis of variance did not show any significant function for these responses at the level of *p* ≤ .05. The first‐order linear model was obtained using the model fitting for acceptance as the function of sprouted buckwheat and sprouted lentil as: Acceptance = 2.27X_1_X_2_–3.79X_1_X_2_(X_1_‐X_2_). In the case of probiotic counts, the first‐order linear mixture model was obtained by model fitting for probiotic counts as the function of sprouted lentil, sprouted buckwheat, and LAB as: Probiotic counts = 0.94X_1_X_2_–0.16X_2_X_3_. According to the combined mixture's design, the optimal condition for beverage formulation showed a buckwheat concentration of 51.96%, lentil concentration of 48.04%, and the lactic acid bacterium of *B. bifidum* as the optimal conditions for beverage formulation. The analysis of variance (ANOVA) results regarding the beverage properties based on combined design (Table 3) noticed that the obtained regression model was significant (*p* < .05). However, the lack‐of‐fit value was insignificant (*p* ≥ .05), suggesting that the suggested model was significantly well fitted. As well, significant correlation degrees were observed between experimental and predicted data especially in terms of total solid values, total phenol, ash content, and the acidity value of the fermented beverages. Formulation under these optimized conditions provided the maximum acidity value of 0.33 (% lactic acid), maximum total solid value of 7.62%, maximum ash value of 0.3%, the highest DPPH percent of 42.3%, the highest phenol content of 29.5 (g/kg), probiotic counts of 8.9 log (CFU ml^−1^), pH of 5.06, and the acceptance score of 3.84. The best formulation of beverage can be set based on each optimum found for each component and each factor applied in the design. The selected formulation of probiotic beverage was prepared and evaluated for validating the predicted values. Based upon the validation experiments, the formulation with optimized levels for different ingredients, including sprouted buckwheat, sprouted lentil and LAB as 52, 48 and *B. bifidum*, respectively, was found most suitable for preparation of sprouted buckwheat/lentil‐based probiotic beverage (Table 4).

**TABLE 3:** ANOVA table showing the variables as linear, quadratic, and interaction terms on each response for probiotic beverage properties

Source	pH		Acidity		Total solid		Ash		Acceptance		DPPH		Total phenol		Probiotic counts	
	SS	*p* ^a^	SS	*p* ^a^	SS	*p* ^a^	SS	*p* ^a^	SS	*p* ^a^	SS	*p* *	SS	*p* *	SS	*p* *
Model	0.66	**.03**	0.04	**.03**	2.11	**<.00**	0.01	**<.00**	2.52	**.00**	84.50	**.02**	127.30	**<.00**	.28	**.01**
Linear Mixture	0.03	.32	0.00	.33	1.95	**<.00**	0.01	**<.00**	1.29	**.00**	81.24	**.00**	127.30	**<.00**	.00	.67
AB	0.13	0.07	0.01	**.04**	0.00	.48			0.81	**.00**	0.01	.96	—	**—**	.13	**.00**
AC	0.04	.27	0.00	.38	0.01	.23	—		—	**—**	0.02	.94	—	**—**	.02	.25
BC	0.44	**.00**	0.00	.64	0.00	.32	—		—	**—**	2.25	.45	—	**—**	.11	**.01**
ABC	0.22	**.03**	0.04	**.03**	0.01	.11			—	**—**	1.94	.48	—	—	.03	.13
AB(A‐B)					0.07	**.00**			0.49	**.01**						
ABC(A‐B)					0.08	**.00**										
Lack of Fit	0.11	.79	0.00	.93		.21		.09		.17		.21		.71		.99
R^2^		.72		.72		.99		.83		.80		.74		.87		.76
Adjusted R^2^		.54		.55		.98		.81		.74		.58		.86		.61
C.V.%		3.37		20.11		.74		4.37		6.99		4.47		4.03		1.17
Std. Dev.		.18		.04		.05		.015		.25		1.90		1.21		.10

*Note*: SS, sum of squares; CV, Coefficient of Variation, A, linear effect of buckwheat; B, linear effect of lentil; C, linear effect of LAB; AB interaction effect of buckwheat (lentil), AC, interaction effect of buckwheat (LAB); BC, interaction effect of lentil (LAB).

a Bold values indicate statistical significance (*p* ≤ .05).

**TABLE 4:** Optimized solutions with predicted and actual experimental values for nondairy probiotic beverage

Solution number Level of ingredients																		
1	Buckwheat	Lentil	LAB	Desirability	pH		Acidity (% lactic acid)		Total solids (%)		Ash (%)		Probiotic counts (log10CFU ml^−1^)		Total phenol (mg GAE/ml)		Acceptance Score	
	52.23	47.77	B.bifidum	0.666	predicted	Exp	predicted	Exp	predicted	Exp	predicted	Exp	predicted	Exp>	predicted	Exp	Predicted	Exp
					5.06	5.2	0.33	0.35	7.63	7.43	0.35721	0.34	8.95	9.1	29.5	30	3.83	3.78

*Note*: Exp: Experimental.

### 3.3 Effect of ingredients' level on pH

The initial pH values of buckwheat and lentil flours were 6.25 and 6.51, respectively. The results of the pH measurements indicate that the fermentation of beverages led to the reduced pH in beverages. Within 24 h of the fermentation, all the beverages reached an average pH value of 5.3, which was significantly different from the pH value measured before the fermentation process (*p* < .05). After 24 h of fermentation, the pH values of the fermented beverages were in the range of 5 to 5.9. As shown in Table 1, after 24 h of the fermentation process, the lowest pH value of 5 related to the treatment number (6) was obtained when *B. bifidum* fermented the beverage made with 75% buckwheat and 25% lentil (*p* ≤ .05). Furthermore, the maximum pH value of 5.9 related to the treatment number (7) was obtained when *B. bifidum* fermented the beverage made with 100% lentil (*p* ≤ .05).

As shown in Table 1, after 24 h of the fermentation process, the pH value of 5.7 related to the treatment numbers (14) and (3) was obtained when *B. bifidum* fermented the beverage made with 100% buckwheat and 50/50% buckwheat/lentil, which were not significantly different. Notably, the pH values of other 10 fermented beverages made with different concentrations of lentil and buckwheat fermented with *L.plantarum* or *B. bifidum* were not statistically different. The increased acidity level with a decrease in pH as fermentation proceed may eliminate the growth of most spoilage and pathogenic microorganisms that cannot withstand such condition, hence making the probiotic nondairy beverages safer for consumption and help to increase their shelf life (Hassan et al. 2012).

### 3.4 Effect of ingredients' level on acidity

Table 1 demonstrates the acidity values of fermented beverages after 24 h of fermentation. The acidity values of the fermented beverages were in the range of 0.11%–0.35% lactic acid. As presented in Table 1, the maximum acidity value belonged to the fermented beverage made with 100% buckwheat (6) or 100% lentil (4) (*p* ≤ .05). Correspondingly, this result shows that acidity was significantly affected by the amount of sprouted buckwheat and lentil flours. As well, the maximum acidity was observed at higher concentrations of these ingredients. This result was similar to the result reported in the study by Sharma et al. (2014). Subsequently, the beverages produced by 100% lentil and the sample made from 50% buckwheat and 50% lentil presented significantly higher acidity values and interestingly, both of them were fermented with *B. bifidum*. The initial acidity values of buckwheat and lentil flours were calculated as 0.08 (% lactic acid) and 0.06 (% lactic acid), respectively. The acidity values obtained for probiotic beverages ranged from 0.10 (% lactic acid) to 0.35 (% lactic acid) (as shown in Table 1). During 24 h of the fermentation, the average acidity value of the beverages reached 0.21 (% lactic acid), which was statistically significantly different from the acidity value measured prior to the fermentation process (*p* < .05). This result indicates that acidity increases after the fermentation of beverages. In comparison with the acidity values obtained with the two LAB strains used in this study, *B. bifidum* tolerated more acidic conditions compared to *L. Plantarum*. In addition, the beverages fermented with *L. plantarum* were more sour beverages compared to the beverages fermented with *B. bifidum*. On the other hand, the results of sensory evaluation confirmed that these sour beverages had a low acceptance score. As expected from the above‐mentioned results, the acidity values of the beverages fermented with *B. bifidum* were higher than the acidity values of those beverages fermented with *L. plantarum*. In the other words, *B. bifidum* was found to be more effective on raising the acidity of beverage in both substrates (buckwheat and lentil) compared to *L. plantarum*. These observations were in agreement with the results found for Iranian white cheese in another study (Zomorodi et al. 2011).

### 3.5 Effect of ingredients' level on ash content

**FIGURE 1:**
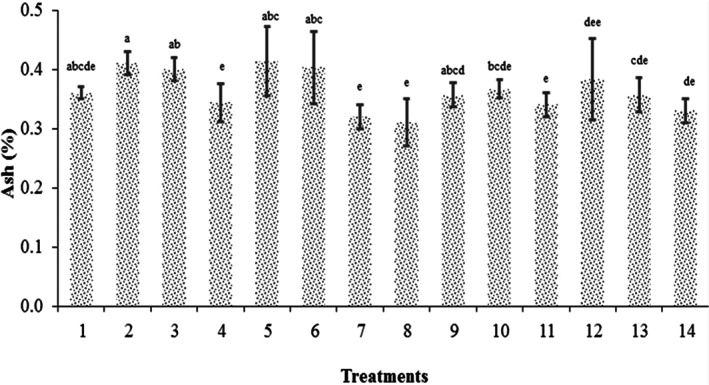
Ash content of the beverages by passing 24 h from the fermentation

The chemical composition analysis showed that the ash values of lentil and buckwheat were 0.11% dm and 3.4% dm, respectively, before sprouting, while the ash contents of sprouted lentil and sprouted buckwheat were 2 ± 0.1 (% dm) and 3.8 ± 0.2 (% dm), respectively. This result was similar to the result reported by Atudorei et al. (2021) that on germination, there was a significant increase (*p* < .05) in ash content for lentil. The significant increase (*p* < .05) in ash content for lentil flour could be due to the increase in phytase activity during germination, which hydrolyzed the bond between the proteins, enzymes, and minerals, to release the minerals (Atudorei et al. 2021). It seems that the ash variation during the germination process depends on the grain type and the germination period (Atudorei et al. 2021). Different studies reported that the ash value presented the highest value after 72 h of germination period, the time used by us for the lentil and buckwheat germination (Atudorei et al. 2021). Correspondingly, this result implies that sprouting the grains increases the ash amounts of the initial raw material used for beverage production. The average ash content of the fermented beverages produced by sprouted grains was 0.35% dm. Figure 1. shows the Ash content of the beverages 24 h after fermentation.

### 3.6 Total phenols and antioxidant activity

**FIGURE 2:**
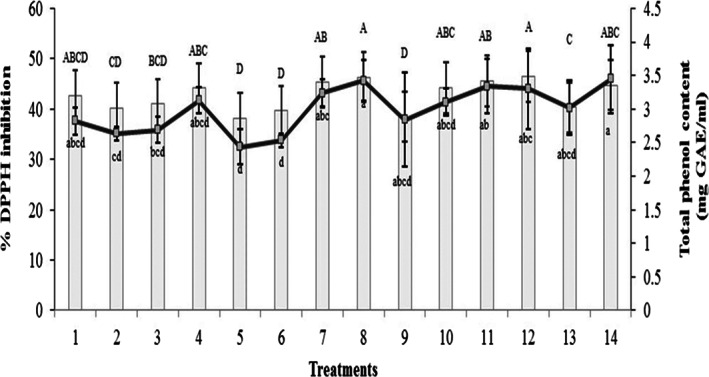
Total phenol content and scavenging activity on DPPH by passing 24 h from the fermentation

The scavenging activity on DPPH radical of buckwheat and lentil was obtained as 19.8% and 25.2%, respectively. The scavenging activity on DPPH after 24 h of fermentation is shown in Figure 2. The scavenging activity on DPPH radicals was found to be affected by the fermentation. The scavenging activity of all beverages was 1.6 and 2.1 fold higher than the raw materials. Total phenol was also affected by this fermentation process. As shown in Figure 2, the average phenol after 24 h of fermentation was 30 (mg GAE/ml). The results of DPPH % and total phenol (mg GAE/ml) were observed to be highly correlated. In case of the two substrates used in this study, important amounts of DPPH were gained when *B. bifidum* was used for fermentation of beverage, showing that the higher antioxidant activity can be obtained in beverages fermented with this bacterium. Additionally, an interesting result of this study is higher amounts of total phenol obtained when using *B. bifidum* for the fermentation of beverage, showing that higher phenol can be obtained in the beverages fermented with this bacterium. The lowest phenol content and lowest DPPH% were obtained for beverages with 100% buckwheat in beverage mixture (treatment number 5). It has been proved that phenolic components are substances with antioxidant activity and they can be a good predictor of this attribute (Velioglu et al. 1998).

### 3.7 Total solid value of the beverages after 24 hours of fermentation

**FIGURE 3:**
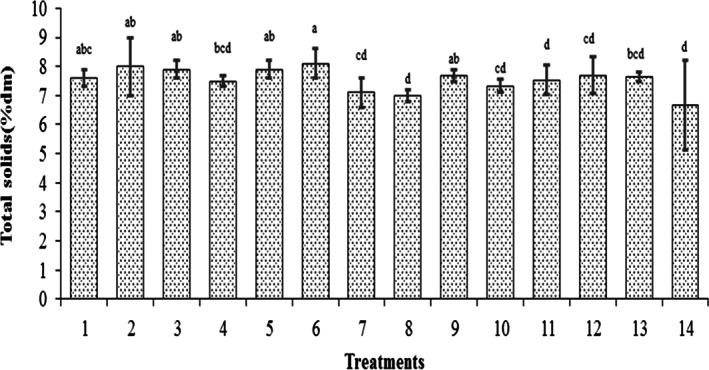
Total solid values of the beverages 24 h after the fermentation

The initial total solid values of buckwheat and lentil flours were 96.8 (% dm) and 97.2 (% dm), respectively. Total solid values of the beverages 24 h after the fermentation are shown in Figure 3. As depicted in Figure 3, the average total solid value of the fermented beverages after 24 h from the fermentation reached 7.55 (%dm). The maximum total solid value (8.1), which was related to beverage sample number 6, was observed in the sample with higher ash content, higher acidity, lower pH, and higher probiotic cell counts. The total solid value of this sample was significantly different from those of other samples (*p* < .05), which exhibited a higher yield of microbial metabolites at the end of the fermentation process. This observation is largely due to the superior bacteria cell population.

### 3.8 Probiotic count after fermentation and during storage

The number of viable cells of *lactobacilli* and *bifidobacteria* on the first day of the experiment were 9.9 and 9.6 log (CFU ml^−1^), respectively, which were over 9 log (CFU ml^−1^). Table 1 figured out the probiotic counts after 24 h from the fermentation. The obtained range of survival rate for LAB at different buckwheat and lentil mixtures applied in the D‐optimal design was from 8.3 to 8.9 log (CFU ml^−1^), indicating the potential health‐promoting properties. During 24 h of the fermentation, the number of viable cells for all the beverages decreased, which reached an average probiotic count of 8.81 log (CFU ml^−1^) that was statistically significantly different from the probiotic count before fermentation (*p* < .05). The reduced probiotic count of the beverages is related to the adaptability of probiotic cells with the substrate.

**FIGURE 4:**
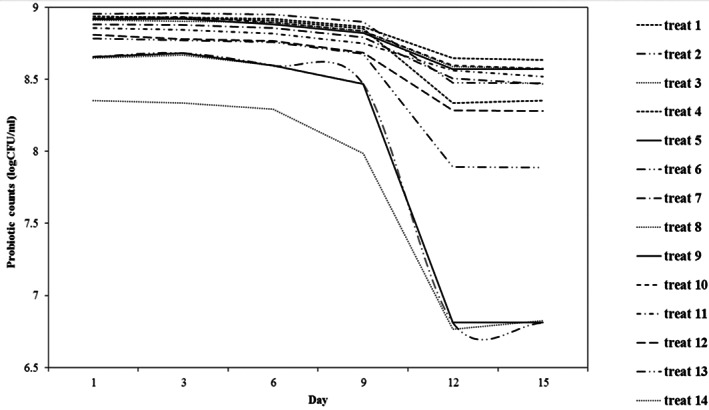
Probiotic population during refrigerated storage

During the storage (Figure 4), the count of probiotics showed no significant decrease until day 6 of the fermentation, except the beverage samples 7, 3, and 5. From the sixth day to the ninth day of the storage period, the count of probiotics showed a slight decrease trend, except the beverages (7, 3, and 5). As shown in Figure 4, until the ninth day of storage, most lines have a gentle slope, but from the ninth day until the twelfth day of storage, the lines are relatively steeper, and the probiotic counts decrease rapidly. Beverages 7, 3, and 5 were the beverage samples fermented with *B. bifidum*, which showed a descending order of probiotic counts from the first day of storage. At the end of the storage (15th day), the beverages contained an average of 8.4 log (CFU ml^−1^) of live lactobacilli cells and 7.8 log (CFU ml^−1^) of viable bifidobacterial cells. The count of viable lactobacilli and bifidobacteria cells on the final day of the experiment was more than 7 log (CFU ml^−1^), indicating the potential health‐promoting properties. The beverages exhibited a high probiotic viability after 15 days of cold storage. The supplementation of the beverages with high fructose corn syrup (HFCS) was the main factor for maintaining the microbial viability at high concentrations (˃10^7^ CFU ml^−1^) during 15 days of the refrigerated storage (4°C). The beverages had the desirable viable count (>10^6^ CFU ml^−1^) of probiotic when kept at 4°C for 15 days, concluding the implementation of the developed beverage at commercial level (Chaturvedi and Chakraborty 2021).

The obtained results were similar to the result reported in the study by Freire et al. (2017), suggesting that beverages made from rice and maize fermented with L. acidophilus and *L. plantarum* supplemented with fructooligosaccharides (FOS) led to the microbial viability of (˃10^7^ CFU ml^−1^) during 28 days of the refrigerated storage (at 4°C).

Certain nondairy plant‐based food beverages, such as pseudocereals and legumes, have been used as successful carriers in delivering probiotics to humans. These food matrices can also enhance the gastrointestinal survival of probiotics, one of the important functional properties that should be fulfilled in providing health benefits for the consumers. Therefore, nondairy plant‐based food products play a significant role in delivering probiotics to humans (Rasika et al. 2021).

### 3.9 Shelf life of the probiotic beverages

**FIGURE 5:**
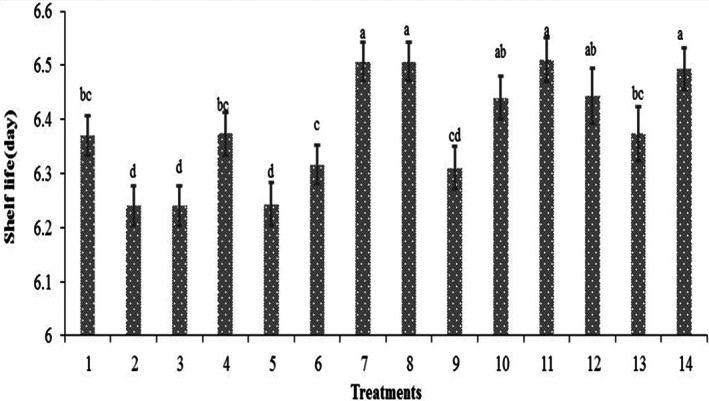
Estimation of the average shelf life of the probiotic beverages

The average shelf life of the probiotic beverages is shown in Figure 5. As shown in this figure, the average shelf life of the probiotic beverages was estimated to be 5 days under the refrigerated storage. Accordingly, during these days, the pH values remained stable above 4.0 ± 0.36 depending on the strain and elongation of the refrigerated period and the number of viable cell counts was above 10^7^ CFU ml^−1^. For these beverages, the fermentation process was done by lactic acid bacteria that converted carbohydrates to alcohol, CO_2_, organic acids, and other secondary compounds that deeply affected the sensory properties of the product, while improving its nutritional value, assuring its safety, and prolonging its shelf life.

Probiotics, in particular lactobacilli and bifidobacteria in nondairy plant‐based food matrices, clearly demonstrated their ability in maintaining adequate viable probiotic numbers (10^6^–10^8^ cfu/ml or g of the carrier food product) during product shelf life (Rasika et al. 2021).

### 3.10 Acceptance of the probiotic beverages

**FIGURE 6:**
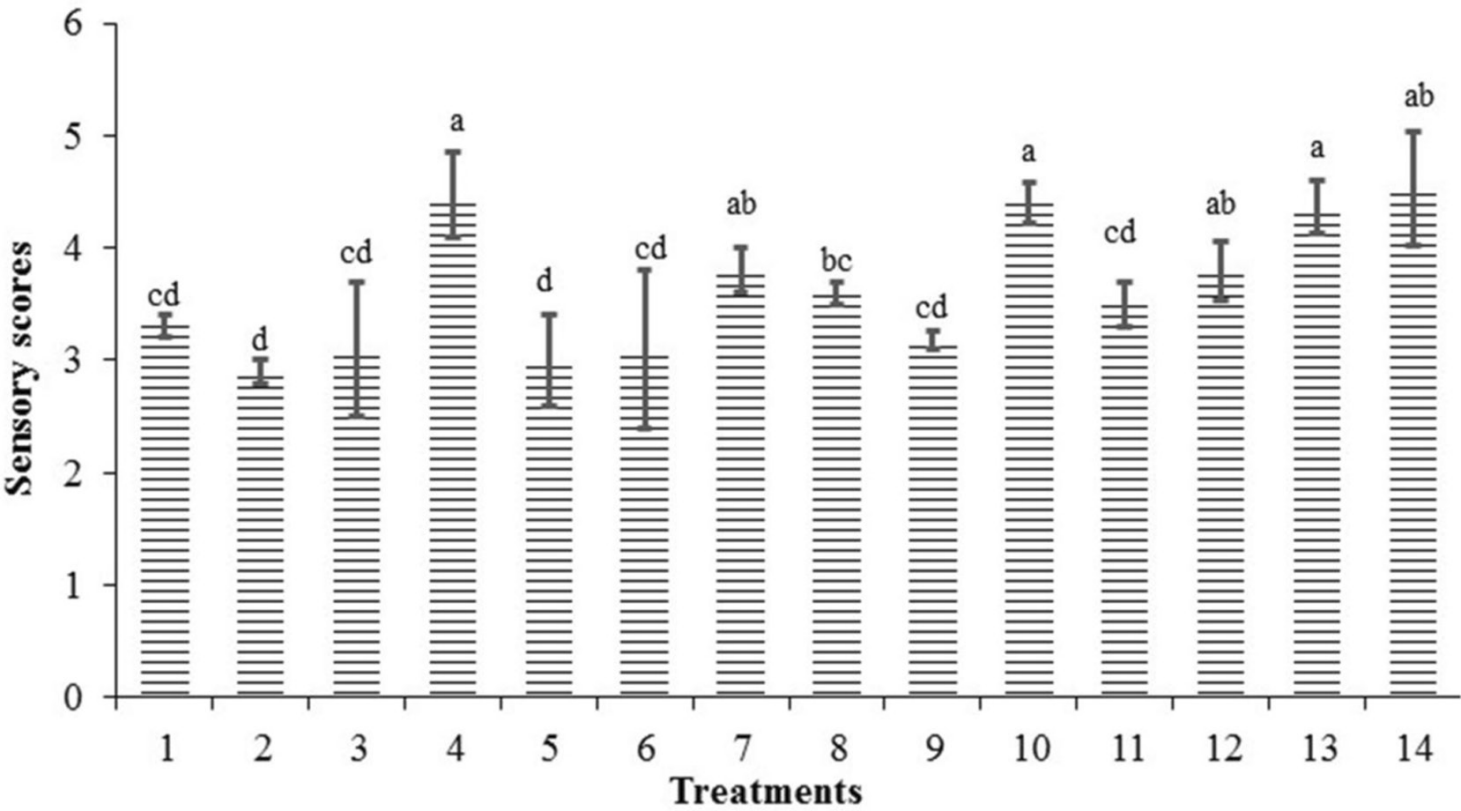
Average acceptance score of the beverages 24 h after fermentation

Sensory properties of probiotic beverages can be affected by interactions between probiotics and food matrices, whereas textures, taste, aroma, and color could be affected by the production of various metabolic compounds. The evaluation of the sensory properties and consumer acceptance of novel probiotic beverages is another important feature needed to be specified during both production and storage processes. The sensory characteristics of nondairy beverages were later studied and the effects of the LAB strains and different substrate produced beverages with different physicochemical characteristics and acceptance rates were observed. The sensory panel average score for the fermented beverages was 4.8 in a 9‐hedonic scale study. The general acceptability of the beverages was good with high scores compared to the unfermented ones, indicating their high marketing potential. The highest sensory scores were obtained for the buckwheat‐based beverages, lentil‐based beverages, and buckwheat–lentil (50:50)‐based beverages (treatment numbers 4, 7, 10, 12, 13, and 14; Figure 6). These beverages also exhibited higher shelf life, higher total phenol content, higher antioxidant activity, high probiotic counts, and mid‐pH values.

Chavan et al. (2018) reported that the fermentation improved the sensory acceptability and the functional characteristics of the several probiotic plant‐based drinks made from germinated and ungerminated barley seeds, finger millet, and moth beans.

Wang et al. (2018) showed that probiotic fermentation enhanced the overall quality of the synbiotic chickpea beverage with acceptable sensory appeal. Dabbagh Moghaddam et al. (2018) studied the production of saffron‐based probiotic beverage by lactic acid bacteria and reported that overall acceptance of the beverage fermented with Lb. Casei (3.8) and Lactococcus lactis (3.9) achieved best scores by pilots and other panelists compared to control sample (4.1).

The buckwheat–lentil (50:50) and buckwheat–lentil (25:75)‐based beverage (treatment numbers 4, 10, and 13) were significantly different from the other fermented beverages in terms of taste, smell, color, mouthfeel, and general acceptability. These samples were the most preferred in terms of the above‐mentioned quality attributes. The unacceptable taste recorded in samples numbered (2 and 5) reduced acceptance score of these beverages. The comments made by most of the panel members indicated a slightly bitter taste and this attributed to the buckwheat flour. These results are close to the result previously reported by other authors where lactic acid fermented beverages with mid‐pH concentrations were preferred (Salmerón 2017). In contrary to our result, the use of higher amounts of buckwheat sprout in bread formulation led to significant negative effects on consumer acceptance (Starowicz et al. 2018).

However, regulating the processing of synbiotic products from the beginning of production to the end of the shelf life is needed for guarding consumer acceptance. For this purpose, several techniques can be used which primarily include fortifying the product with prebiotics and adding probiotic microcapsules (Dias et al., 2018; Chan et al., 2020).

## 4 CONCLUSION

In the present study, single‐culture fermentation was prepared in mixed and single pseudocereal–legume substrates and then evaluated. The results of the composition analysis of grain flour showed the total solid value of buckwheat and lentil flours as 96.8% and 97.2%, respectively. The dietary fiber content of buckwheat and lentil flours was 7 (% dm) and 13.43 (% dm), respectively. The pH and acidity values of buckwheat flour were calculated as 6.25 and 0.08 (% lactic acid), respectively. The pH and acidity values of lentil flour were 6.51 and 0.06 (% lactic acid), respectively. According to the results of beverage properties' modeling, the first‐order linear mixture model was obtained by model fitting for pH, acidity, total solids, and probiotic counts as the function of sprouted buckwheat, sprouted lentil, and LAB. The first‐order linear model was obtained using the model fitting for acceptance as the function of sprouted buckwheat and lentil. The linear mixture model for the ash content, DPPH, and total phenol was significant, although analysis of variance did not show any significant function for these responses at the level of *p* ≤ .05. According to the combined mixture's design, the optimal condition for beverage formulation showed a buckwheat concentration of 51.96%, lentil concentration of 48.04%, and the lactic acid bacterium of B. bifidum as the optimal conditions for beverage formulation. Formulation under these optimized conditions provided the maximum acidity value of 0.33 (%lactic acid), maximum total solid value of 7.62%, maximum ash value of 0.3%, the highest DPPH percent of 42.3, the highest phenol content of 29.5 (g/kg), probiotic counts of 8.9 log (CFU ml‐1), the pH of 5.06, and the acceptance score of 3.84. The acidity values of fermented beverages after 24 h of fermentation were in the range from 0.11% to 0.35% lactic acid. During 24 h of the fermentation, the average acidity value of the beverages reached 0.21 (% lactic acid), which was statistically significantly different from the acidity value measured prior to the fermentation process. The chemical composition analysis showed that the ash values of lentil and buckwheat were 0.11% dm and 3.4% dm, respectively, before sprouting, while the ash contents of sprouted lentil and sprouted buckwheat were 2 ± 0.1 (% dm) and 3.8 ± 0.2 (% dm), respectively. The average ash content of the fermented beverages produced by sprouted grains was 0.35% dm. The scavenging activity on DPPH radical of buckwheat and lentil was obtained as 19.8% and 25.2%, respectively. As shown in Figure 2, the average phenol after 24 h of fermentation was 30 (mg GAE/ml). The initial total solid values of buckwheat and lentil flours were 96.8 (% dm) and 97.2 (% dm), respectively. The average total solid value of the fermented beverages after 24 h from the fermentation reached 7.55 (% dm). The maximum total solid value (8.1) was related to the beverage with higher ash content, acidity, probiotic cell counts, and lower pH. The number of viable cells of lactobacilli and bifidobacteria on the first day of the experiment were 9.9 and 9.6 log (CFU ml^−1^), respectively. The obtained range of survival rate for LAB at different buckwheat and lentil mixtures applied in the D‐optimal design was from 8.3 to 8.9 log (CFU ml^−1^), indicating the potential health‐promoting properties. At the end of the storage, the beverages contained an average of 8.4 log (CFU ml^−1^) of live lactobacilli cells and 7.8 log (CFU ml^−1^) of viable bifidobacterial cells. The average shelf life of the probiotic beverages was estimated to be 5 days under the refrigerated storage. The sensory panel average score for the fermented beverages was 4.8 in a 9‐hedonic scale study. The buckwheat–lentil (50:50) and buckwheat–lentil (25:75)‐based beverage was significantly different from the other fermented beverages in terms of sensory properties.

## CONFLICT OF INTEREST

The authors declare that they have no conflict of interest.

## ETHICS STATEMENT

This study does not involve any human or animal testing.

## Data Availability

The data that support the findings of this study are available from the corresponding author upon reasonable request.
